# Fgf2 improves functional recovery—decreasing gliosis and increasing radial glia and neural progenitor cells after spinal cord injury

**DOI:** 10.1002/brb3.172

**Published:** 2014-01-13

**Authors:** Yona Goldshmit, Frisca Frisca, Alexander R Pinto, Alice Pébay, Jean-Kitty K Y Tang, Ashley L Siegel, Jan Kaslin, Peter D Currie

**Affiliations:** 1 Australian Regenerative Medicine InstituteEast Melbourne, VIC, Australia; 2 Centre for Eye Research Australia & Royal Victorian Eye and Ear HospitalEast Melbourne, VIC, Australia; 3 Department of Ophthalmology, The University of MelbourneEast Melbourne, VIC, Australia

**Keywords:** Astroglia, GFAP, nestin, Pax6, progenitors, regeneration, Sox2, spinal cord injury

## Abstract

**Objectives:**

A major impediment for recovery after mammalian spinal cord injury (SCI) is the glial scar formed by proliferating reactive astrocytes. Finding factors that may reduce glial scarring, increase neuronal survival, and promote neurite outgrowth are of major importance for improving the outcome after SCI. Exogenous fibroblast growth factor (Fgf) has been shown to decrease injury volume and improve functional outcome; however, the mechanisms by which this is mediated are still largely unknown.

**Methods:**

In this study, Fgf2 was administered for 2 weeks in mice subcutaneously, starting 30 min after spinal cord hemisection.

**Results:**

Fgf2 treatment decreased the expression of TNF-a at the lesion site, decreased monocyte/macrophage infiltration, and decreased gliosis. Fgf2 induced astrocytes to adopt a polarized morphology and increased expression of radial markers such as Pax6 and nestin. In addition, the levels of chondroitin sulfate proteoglycans (CSPGs), expressed by glia, were markedly decreased. Furthermore, Fgf2 treatment promotes the formation of parallel glial processes, “bridges,” at the lesion site that enable regenerating axons through the injury site. Additionally, Fgf2 treatment increased Sox2-expressing cells in the gray matter and neurogenesis around and at the lesion site. Importantly, these effects were correlated with enhanced functional recovery of the left paretic hind limb.

**Conclusions:**

Thus, early pharmacological intervention with Fgf2 following SCI is neuroprotective and creates a proregenerative environment by the modulation of the glia response.

Please note that an editorial related to this article, “The role of FGF2 in spinal cord trauma and regeneration research,” doi: 10.1002/brb3.207, can be found here, also published in *Brain and Behavior*.

## Introduction

In mammals, a major barrier for axonal regeneration after spinal cord injury (SCI) is the formation of the glial scar at the lesion. The glial scar is composed of astrocytes, which are triggered in response to extrinsic signals to activate and proliferate to generate a dense network of hypertrophic stellate cells that form an impenetrable barrier to the regrowth of damaged axons. Thus, one therapeutic strategy could be to improve the environmental conditions at the lesion site post-SCI to better support neuronal survival and axonal regrowth. Neurotrophic factors are good candidates to be examined due to their supportive role during developmental neurogenesis. In addition to improving environmental signals, therapies need to target the cells that are already present at the injury site as these can play crucial roles in either supporting or blocking regeneration. For example, induction of radial glia-like and neuronal progenitor cells, which during development serve as scaffolds to support neuronal migration and give rise to neurons, may improve regeneration. It has been shown that in the adult brain, bipolar or unipolar glial fibrillary acidic protein (GFAP)-expressing cells at the subependymal zone of the lateral ventricle or the hippocampus dentate gyrus are the neural progenitor sources (Garcia et al. 2004). These cells also express nestin, which is not expressed by stellate astrocytes but is found on the majority of neuronal progenitor cells in the adult brain (Mignone et al. 2004). After rodent SCI, injected radial glia can be neuroprotective and improve functional recovery (Hasegawa et al. 2005; Chang et al. 2009). Studies in optic nerve regeneration in young rats suggested that not only neonatal astrocytes but also astrocyte-like glia in older rats improve and support axonal regeneration (Dyson et al. 1988; Harvey and Tan 1992); however, the cellular process that guides this regeneration remains unclear. In addition, neuronal stem cells expressing transcription factor, such as Sox2, are also increased after SCI (Lee et al. 2012; Rodriguez-Jimnez et al. 2012). With the right factors, local and reactive astrocytes in the gray and white matter may be turned into radial glia or neuronal progenitors that better support neurogenesis and regeneration.

The application of fibroblast growth factor (Fgf)2 has been shown to promote functional recovery after brain injury (Dietrich et al. 1996; McDermott et al. 1997), stroke (Kawamata et al. 1996, 1997), or SCI (Lee et al. 1999; Rabchevsky et al. 1999; Yan et al. 2000). In SCI the recovery is thought to be due to Fgf promoting neuronal survival (Teng et al. 1998, 1999), angiogenesis (Kang et al. 2013), and causing a reduction in injury volume (Lee et al. 1999; Rabchevsky et al. 1999). Several therapies that claim regenerative effects after transplanting specific cells also contain a cocktail of factors including Fgf1 and Fgf2 (Meijs et al. 2004; Kuo et al. 2011; Guzen et al. 2012; Lu et al. 2012), and thus, the proregenerative capacity attributed to transplanted cells may in fact partially be due to the proregenerative effects of Fgf signaling. Furthermore, Fgf are currently in clinical trials in human patients with cervical SCI (Wu et al. 2008). Therefore, it is important to understand the cellular and molecular mechanism by which Fgf contributes to regeneration.

We recently demonstrated that Fgf signaling plays a crucial role in glial cell differentiation and morphogenesis that is required for regeneration after SCI in zebrafish (Goldshmit et al. 2012). After SCI in adult zebrafish, radial glia in the central canal form bridges to support axonal regeneration through the lesion. Moreover, we and others have demonstrated that in zebrafish radial glia at the injury site generate new neurons during regeneration (Reimer et al. 2008; Hui et al. 2010; Kroehne et al. 2011). Therefore, we examined if Fgf2 mediates gliogenesis and glial morphogenesis to attenuate scar formation after SCI in the mammalian spinal cord in a similar way to that observed during spinal cord regeneration in zebrafish.

Our study shows that Fgf2 application after mammalian SCI does influence glial cell activation, generating a proregenerative radial/progenitor-like state. Fgf2 increases the presence of progenitor cells at the lesion site in both gray and white matter. Application of Fgf2 increases the number of cells expressing progenitor markers, such as Pax6, nestin, and Sox2, at the lesion site short term after injury. Fgf2 also influences glial morphology to become bipolar and support axonal regeneration rather than the hypertrophic cells evident during reactive gliosis and glial scar formation that are inhibitory to axonal regeneration. Taken together, our study demonstrates that Fgf2 can orchestrate proliferating astrocytes at the lesion site of a mammal to give rise to glia progenitor cells rather than reactive astrocytes that form scar tissue.

## Materials and Methods

### Mice

Adult (2 months) male C57BL/6 mice were used. All procedures were approved by Monash University Animal Ethics Committee in accordance with the requirements of the National Health and Medical Research Council of Australia. In total 70 mice were used in the study.

### Spinal cord hemisections

As described (Goldshmit et al. 2004), mice (20–30 g) were anesthetized with ketamine (100 mg/kg) and xylazine (16 mg/kg) in phosphate buffered saline (PBS) injected intraperitoneally. The spinal cord was exposed at the low thoracic to high lumbar area. After laminectomy, a complete left hemisection was made at T12 and the overlying muscle and skin were sutured. Mice were randomly assigned to the control-PBS or Fgf2 injection groups and allowed to survive for 2 days to 7 weeks postinjury.

### BrdU application

BrdU (100 *μ*L of 15 mg/mL; Sigma-Aldrich, Castle Hill, NSW, Australia) was injected intraperitoneally 0, 2, 4, and 6 days after lesion.

### Fgf2 application

PBS (80 *μ*L) or human Fgf2 (50 *μ*g/mL) (Miltenyi Biotec GmbH, Bergisch Gladbach, Germany, total dose 135 *μ*g/kg [Yan et al. 2000]) were subcutaneously injected 30 min and every second day after SCI. The first injection was delivered at the back under the skin of the operated area just above the lesion site, whereas the other injections were performed subcutaneously at the abdominal area next to the left hind limb, where the secession is impaired in order to prevent unnecessary pain for the animal.

### Behavioral analyses

Two examiners tested mice before and 24 h to 5 weeks after SCI. In the tests, the performance of the mice is individually evaluated before and after the injury. *Horizontal grid walking* (Goldshmit et al. 2004, 2011): After 2 min of free walking, missteps (normalized to total number of steps taken by the left hind limb) were quantified. *Open-field locomotion score*: Evaluated for 3 min using the modified Basso–Beattie–Bresnahan (mBBB) scoring system of 20 points (PBS *n* = 11, Fgf2 *n* = 13) (Li et al. 2006).

### Anterograde axonal tracing

Axonal regeneration was examined using anterograde tracing (PBS control *n* = 8, Fgf2 *n* = 10 for 7-week experiments; PBS control *n* = 4, Fgf2 *n* = 4 for 4-month experiments). Seven weeks or 4 months after SCI, tetramethylrhodamine dextran (TMRD) (“Fluoro-Ruby,” MW 10,000 kD; Molecular Probes, Grand Island, NY) was injected into the spinal cord at the level of the cervical enlargement, ipsilateral to the lesion as described (Goldshmit et al. 2004). After 7 days, mice were perfused with PBS, then 4% paraformaldehyde (PFA). Spinal cords were removed and postfixed for 1 h in cold 4% PFA followed by 20% sucrose in PBS overnight at 4°C. Longitudinal (horizontal) serial cryostat sections were cut (50 *μ*m) and slides were imaged using fluorescence and confocal microscopy. Labeled axons in the white matter were quantified 0–100 *μ*m proximal to the lesion site at 400×. Photomontage of the regenerating axons was taken on a laser scanning confocal microscope, Zeiss LSM510 (Carl Zeiss, Sydney, NSW, Australia).

### Immunohistochemistry

Cryostat sections (20 *μ*m) were stained using standard immunohistochemistry. Primary antibodies: rabbit anti-GFAP (1:1000; Dako, Noble Park, VIC, Australia), mouse anti-GFAP (1:1000; Invitrogen, Mulgrave, VIC, Australia), rabbit antidoublecortin (DCX) (1:400; Cell Signaling, Arundel, Qld, Australia), rabbit anti-Pax6 (1:300; Covance), mouse antinestin (1:300; Cell Signaling), mouse anti-*β*-Tubulin (1:1000; Promega, Alexandria, NSW, Australia); mouse anti-BrdU (1:400; Roche, Hawthorn, VIC, Australia), rat anti-BrdU (1:200; Abcam, Cambridge, MA), mouse anti-HuC/D (1:250; Invitrogen), mouse anti-chondroitin sulfate proteoglycan (CSPG) (clone CS-56) (1:200; Sigma), rat anti mouse-CD11b (1:200; Invitrogen), and mouse anti-Sox2 (1:200, Sigma). Secondary antibodies: Alexa Fluor 488, 568, or 633; 1:1000 (Invitrogen). Nuclei were visualized with 4′,6-diamidino-2-phenylindole (DAPI) (Sigma). Antigen retrieval was performed by incubation in 2-mol/L HCl for 15 min (BrdU) or 1-mol/L Tris-HCl (pH:8.0) at 90°C for 20 min (HuC/D).

### Flowcytometry analysis of spinal cord tissue

After isolation of damaged spinal cords (1 mm from each side of the injury; *n* = 6 animals from each group), single cell suspensions were prepared using the “rapid protocol” as described previously (Pinto et al. 2013). Flowcytometry analysis was conducted as previously described (Pinto et al. 2013), by immunostaining prepared single cell suspensions with rat antimouse CD45 (clone 30-F11; eBioscience, Kensington, SA, Australia), CD11b (clone M1/70; BioLegend, Karrinyup, WA, Australia), and CD14 (clone Sa2-8; eBioscience) antibodies. Flowcytometry data were analyzed using FlowJo 7.6.4 software (Ashland, OR).

### qPCR of spinal cord tissue

Spinal cord tissue was dissected (*n* = 4 per group) 3 mm on either side of the lesion and homogenized by a polytron homogenizer (Kinematica, Bohemia, NY) in 1-mL TRI reagent according to manufacture instruction (TRI reagent; Sigma), Extracted RNA from spinal cords was reverse transcribed (Superscript III RT kit; Invitrogen) and analyzed by quantitative polymerase chain reaction (qPCR) performed using TaqMan Universal master mix (Applied Biosystems, Foster City, CA) and the 7900HT fast real-time PCR system (Applied Biosystems). TaqMan gene expression assays were used for detecting mouse *spry4* (Mm00442345_m1), *gfap* (Mm01253033_m1), and tumor necrosis factors (*tnf*)-*α* (Mm00443260_g1) (Applied Biosystems). Using the comparative (CT) method (ΔΔCT), mRNA levels were normalized against levels of glyceraldehyde-3-phosphate dehydrogenase (*gapdh*) mRNA (TaqMan gene expression assay (Mm99999915_g1)) with the control used as reference.

### Cell counting

BrdU-, Pax6-, GFAP-, DCX-, HuC-, CSPG-, Sox2-positive cells were quantified in a 200 *μ*m^2^ box at the lesion, in every third serial longitudinal 20-*μ*m section. The GFAP or CS-56 density was measured in 409 images using Image J (Wayne Rasband, National Institutes of Health) and averaged was calculated from counting at least five boxes per section; from five sections per spinal cord. Number of primary GFAP processes extending from a cell with DAPI-stained nucleus was counted from same images used for GFAP density. Results are presented as percentage of each field showing GFAP expression. Traced axons were counted 100 *μ*m proximal to the lesion from at least 10 sections/spinal cord, in 50-*μ*m sections.

### Microscopy

Sections were imaged by fluorescence microscopy using a Axioplan Z1 (Zeiss, Germany) epifluorescence microscope. Photomicrographs (1300 × 1030 dpi) were obtained with 2.5× and 5× Plan-Neofluar (Zeiss, Germany) objectives, and acquired using a AxioCam (Zeiss, Germany) digital camera using AxioVision software (v. 4.4; Zeiss, Germany). For colocalization analyses, optical sections were acquired with the Apotome module and a 40× objective. Z-stack photomontage of axonal tracing was done using confocal microscope Zeiss 710. Images were sized using Adobe Photoshop 11 and Illustrator 14.

### Statistical analysis

**Figure 1:**
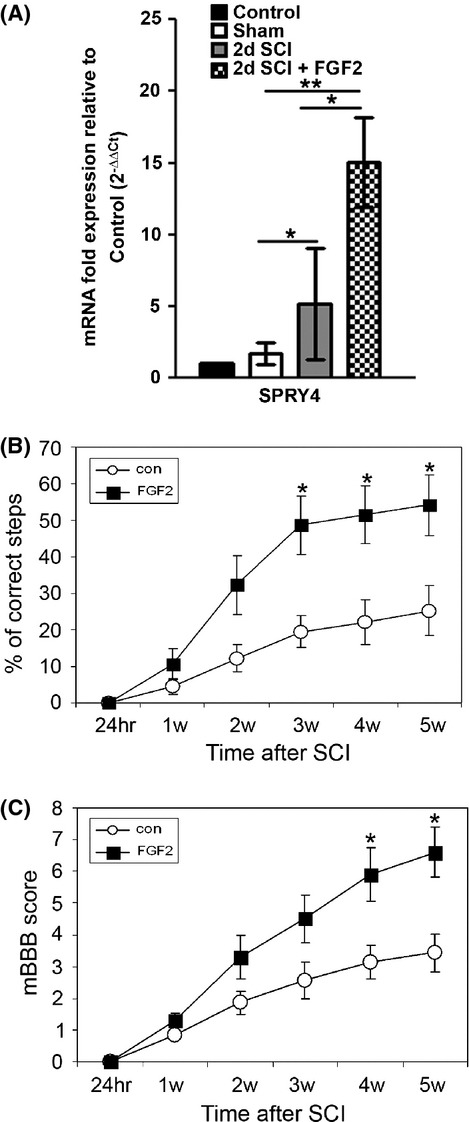
Fgf2 injections improve motor function after SCI. Fgf2 treatment increases *spry4* (A) 2 days after SCI as shown by qPCR (con *n* = 2; sham *n* = 2; SCI *n* = 5; SCI positive Fgf2 *n* = 4). (B) Grid walking (mean ± SEM *P < 0.05) and (C) mBBB score (*P < 0.05) improved significantly 3 or 4 weeks after Fgf2 treatment. Results are mean ± SEM (PBS *n* = 11, Fgf2 *n* = 13). Fgf, fibroblast growth factor; SCI, spinal cord injury; qPCR, quantitative polymerase chain reaction; mBBB, modified Basso–Beattie–Bresnahan; PBS, phosphate buffered saline.

**Figure 2:**
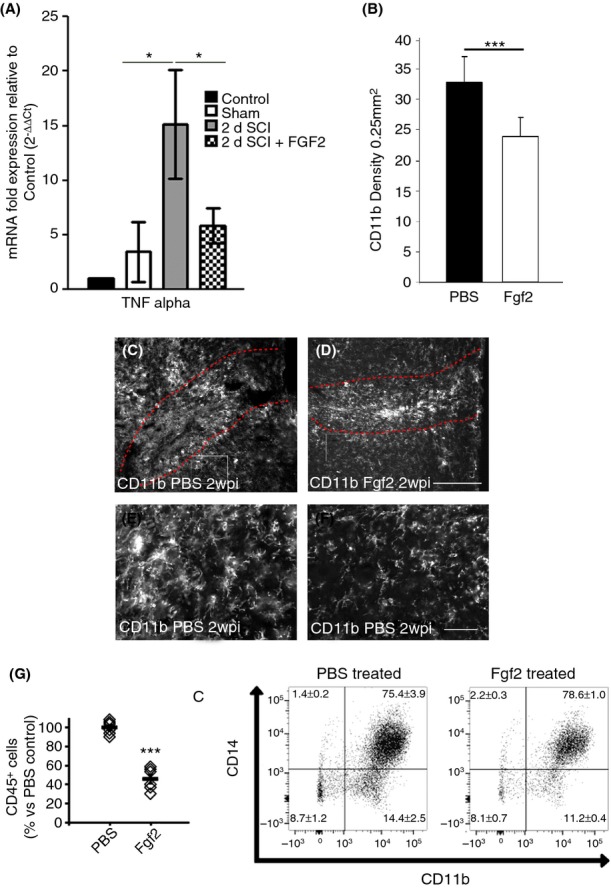
Fgf2 injections decrease the inflammatory response at the lesion site. Fgf2 decreased *tnf-α* mRNA (A) 2 days after SCI as shown by qPCR (control intact *n* = 2; sham operated *n* = 2; SCI *n* = 5; SCI +Fgf2 *n* = 4). SCI, CD11b immunostaining in PBS-control–treated mice (C, E enlargement) compared to reduced levels in Fgf2-treated mice (D, F enlargement). (B) Quantitative analysis of the reactive microglia marker CD11b from 0.25 mm^2^ areas adjacent the lesion site. (G) Flow cytometry analysis of total cells isolated from spinal lesions by staining for the common leukocyte marker CD45, common myeloid marker CD11b, and the monocyte/macrophage marker CD14. Results are mean ± SEM (*n* > 5 in each group; ***P < 0.001, two-tailed *t*-test, 95% confidence). Scale bar in C and D is 200 *μ*m; E and F is 50 *μ*m. Fgf, fibroblast growth factor; SCI, spinal cord injury; qPCR, quantitative polymerase chain reaction; PBS, phosphate buffered saline.

**Figure 3:**
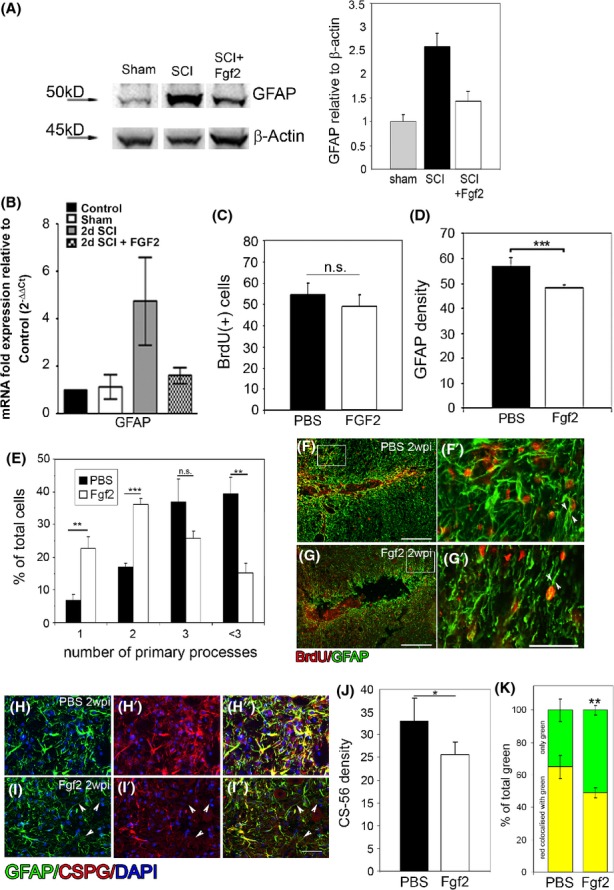
Fgf2 decreases astrocyte reactivity at the lesion site. Seven days after SCI, (A) western blot analysis shows increase in GFAP protein level after injury compared to sham operated. Fgf2 decreases GFAP protein levels after SCI (*n* = 3). Two days after SCI (B), Fgf2 decreased *GFAP* mRNA as shown by qPCR (con *n* = 2; sham *n* = 2; SCI *n* = 5; SCI +Fgf2 *n* = 4). Two weeks after SCI (C), no difference in BrdU-positive cells at the lesion site between PBS and Fgf2 treatments, although (D) GFAP density is significantly reduced after Fgf2 treatment (**P < 0.01). (E) Quantitation of the number (mean ± SEM) of primary processes extending from the cell body in control (PBS) and Fgf2 treatment (*n* = 6 in each group; **P < 0.01, ***P < 0.001, n.s. = nonsignificant) shows an increased number of cells with bipolar morphology in FGF2-treated mice. (F) Astrocytes (GFAP+ green) proliferate (BrdU+ red) in PBS-treated mice (*n* = 5) and exhibit thick multiprocess morphology (G′). In Fgf-treated mice (*n* = 5) (G), proliferating astrocytes (GFAP+/BrdU+) exhibit a bipolar morphology (G′). Immunostaining for CSPGs (J) 2 weeks after lesion revealed that their secretion was diminished in the Fgf2-treated mice (*P < 0.05) and (K, H–H′′, and I–I′′) some GFAP-positive staining dose not labeled with CSPGs (**P < 0.01 in K; I–I′′; arrowheads). Results are mean cell number in the field ±SEM. Scale bars in F and G are 200 *μ*m, in F′, G′, H–H′′ and I–I′′ are 50 *μ*m. Fgf, fibroblast growth factor; SCI, spinal cord injury; qPCR, quantitative polymerase chain reaction; PBS, phosphate buffered saline; CSPGs, chondroitin sulfate proteoglycans.

**Figure 4:**
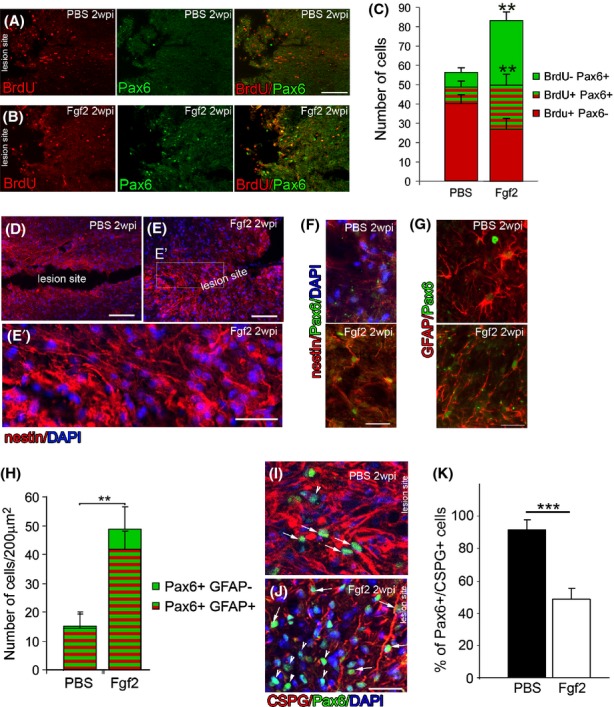
Fgf2 injections increase the number of radial/progenitor-like cells at the lesion site. Two weeks after SCI (A), Pax6 expression at the lesion site in PBS control is very low (*n* = 5). (B) In contrast, many Pax6-positive cells are observed at the lesion site after Fgf2 treatment, many of which are BrdU positive (*n* = 5). (C) The Pax6-positive/BrdU-positive cells are significantly increased in Fgf2-treated mice (**P < 0.001). (D) Compared to PBS mice (E), nestin expression is stronger in the lesion of Fgf2-treated mice, where nestin-positive processes can be seen perpendicular to the lesion (E′). (F) In contrast to PBS control (upper panel), in Fgf2-treated mice, these nestin+ cells colabel with Pax6. (G) In contrast to PBS control that show multipolar morphology and low levels of Pax6 on high GFAP-expressing cells (upper panel), after Fgf2 treatment, these Pax6+ cells also colabel with GFAP, which reveals their polarized morphology (lower panel). (H) The Pax6-positive/GFAP-positive cells are significantly increased in Fgf2-treated mice (**P < 0.001). (I) In PBS control, majority of Pax6-positive cells at the lesion site were surrounded by high levels of CSPG. (J) in Fgf2-treated animals, many of the Pax6-positive cells are not surrounded by CSPGs (K) quantitation of double-positive cells for Pax6, and CSPGs reveal a significant decrease after FGF2 treatment. Results are mean ± SEM (*n* = 5 in each group; ***P < 0.001, two-tailed *t*-test, 95% confidence). Scale bars in A, B, D, and E are 200 *μ*m, in C′ are 10 *μ*m, and in E′, F, G, I, and J are 50 *μ*m. Fgf, fibroblast growth factor; SCI, spinal cord injury; PBS, phosphate buffered saline; CSPGs, chondroitin sulfate proteoglycans.

Significance was evaluated using two-tailed *t*-test with 95% confidence when comparing two parameters in data presented in Figures 2B and G, 3C–E and J–K, 4C, H, and K, 6C and F, 7C, D and E, or one-way analysis of variance (ANOVA) followed by the Tukey test for multiple comparisons with *α* = 0.001 in Figures 1A, 2A, 3A and B (*P < 0.05, **P < 0.001).

The nonparametric Mann–Whitney *U*-test was used to assess significance of differences in the behavioral analysis (Fig. 1B and C, *P < 0.05). Data are expressed as mean ± standard error of the mean (SEM).

## Results

### Fgf2 activates Fgf signaling and promotes functional recovery

In order to examine whether subcutaneous Fgf2 injections can activate Fgf signaling within the spinal cord, mRNA levels of the Fgf downstream target gene *Spry4*, which we have previously shown to be expressed in a mouse spinal cord (Goldshmit et al. 2012), were quantified using qPCR, 2 days after SCI. *Spry4* mRNA levels were significantly increased at the lesion and expression levels were further augmented following Fgf2 injections (Fig. 1A). Functional recovery, assessed up to 5 weeks after SCI, significantly improved following Fgf2 treatment after SCI. There were fewer missteps of the left hind limb during grid walking (Fig. 1B) and significant functional improvement based on the mouse modified open-field behavior test (mBBB scale; Li et al. 2006) (Fig. 1C). Our results in behavioral improvement are in agreement with other studies in rodents (Lee et al. 1999; Rabchevsky et al. 1999; Kojima and Tator 2002). Thus, the Fgf2 injection regime increased Fgf signaling at the lesion site and resulted in improved functional recovery.

### Fgf2 decreases inflammation and astrocyte reactivity at the lesion site

We next assessed which cellular and molecular processes Fgf2 signaling regulates during recovery after SCI. Excessive inflammation and reactive gliosis are detrimental to regeneration after SCI. Therefore, we examined the expression of the proinflammatory factor *tnfα* and activation of microglia/macrophages after SCI and Fgf2 treatment.

qPCR analysis demonstrated that the expression of *tnfα* expression was significantly decreased after Fgf2 treatment (Fig. 2A). This was followed by decreased microglia/macrophage activation, measured by CD11b density, 2 weeks after SCI (Fig. 2B–F). To undertake a more detailed analysis for leukocyte infiltration, we conducted flow cytometry analysis of total cells isolated from spinal lesions by staining for the common leukocyte marker CD45, common myeloid marker CD11b, and the monocyte/macrophage marker CD14. We found that Fgf2 treatment reduced the total number of leukocytes (CD45^+^ cells) infiltrating the injury lesion, which predominantly comprised monocyte/macrophages (CD11b^+^CD14^+^) (Fig. 2G). Thus, Fgf2 moderates the inflammatory response at the lesion site. This decreased levels of TNF*α* and inflammatory cell infiltration in Fgf2-injected animals may decrease the number of reactive astrocytes as suggested in studies in other neurotrauma models in vitro (Tzeng et al. 1999; Toyooka et al. 2011). To study astrocytic activation, we first examined levels of *gfap* mRNA and protein upregulation after injury with or without Fgf2 treatment (Fig. 3A and B). qPCR for *gfap* mRNA 2 days after SCI and western blot analysis for protein levels 7 days after SCI show that in both assays Fgf2 tends to decrease levels of GFAP in the spinal cord.

The vast majority of BrdU-positive cells around the lesion at this point were GFAP positive in both groups (95 ± 2.5%, PBS-control; 91.5 ± 4.0%, Fgf2). Quantitation of astrocytic proliferation (by BrdU) showed no difference by Fgf2 treatment (Fig. 3C). However, Fgf2 treatment reduced the reactivity of these astrocytes. The density of GFAP immunoreactivity around the lesion was significantly lower in Fgf2-treated mice (Fig. 3D). This is in part due to astrocytes in Fgf2-treated mice exhibiting fewer processes than PBS-control mice (Fig. 3E). Additionally, the GFAP-positive processes in the PBS control mice seemed qualitatively thicker compared to the Fgf2-treated mice (Fig. 3F′ and G′, arrowheads).

Thus, Fgf2 treatment does not alter astrocyte proliferation in vivo, but instead decreases the reactivity of astrocytes as quantified by GFAP density, number of primary processes, and the trend observed in the mRNA and protein levels.

Reactive astrocytes are known to produce and express CSPGs at the injury site. CSPGs are inhibitory to axonal regeneration (Jones et al. 2003; Silver and Miller 2004). We found that the density of CSPG expression is significantly lower in the Fgf2-treated mice (Fig. 3J), and GFAP-positive/CSPG-negative processes were significantly increased (Fig. 3K; PBS, 34.9 ± 6.9; Fgf2, 50.8 ± 3.02; **P < 0.01) in the Fgf2-treated mice (Fig. 3H–H′′ and I–I′′, arrowheads). This may suggest that the scarring environment after Fgf2 treatment is less severe and the astrocytes reactivity is reduced.

### Fgf2 mediates proliferation of radial glia at the lesion site

Two weeks after SCI, Pax6 expression, which is an important functional indicator of neurogenic radial glia (Heins et al. 2002), was significantly increased in Fgf2-treated compared to PBS-control mice (55.9 ± 5.4 cell/field; 16.2 ± 2.7 respectively, Fig. 4A–C). This suggests that as well as mediating glial cell morphology, Fgf2 stimulates proliferating astrocytes to regain characteristics of neurogenic radial glia. While the total number of BrdU-labeled cells remains comparable (50.2 ± 7.7 cells/field in Fgf2, 48.9 ± 3.5 control), significantly more proliferative glia express Pax6 within the injured spinal cord of Fgf2-treated animals (23.1 ± 5.4 Fgf2; 8.9 ± 2.7 cells/field control).

These Pax6-positive cells also expressed other markers characteristic of radial glia and neural progenitor cells such as nestin and Sox2. After Fgf2 treatment, Pax6-positive/nestin-positive cells around the lesion site displayed long bipolar processes arranged perpendicularly to the plane of hemisection (Fig. 4D–F). Furthermore, the proportion of GFAP and Pax6 double-positive expressing cells increased significantly after Fgf2 treatment (Fig. 4G–H). Some of these cells possessed bipolar (Fig. 4G, lower panel) rather than the multipolar morphology of reactive astrocytes in PBS-control (Fig. 4G, upper panel). Furthermore, many of the Pax6-positive cells do not colabel with CSPGs after Fgf2 treatment, suggesting that these cells lose the characteristics of reactive astrocytes (Fig. 4I–K).

### Fgf2 mediates glial bipolar morphology at the lesion site to support neurite elongation and axonal regeneration

**Figure 5:**
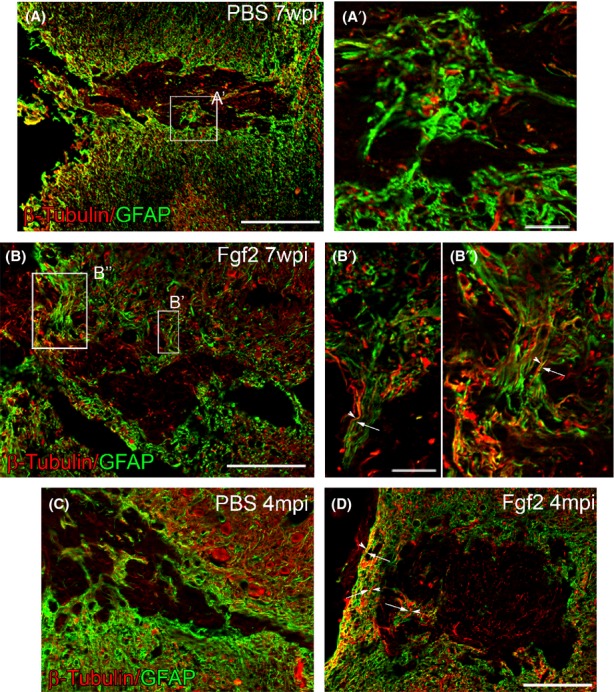
Fgf2 mediates glial bridge formation. Seven weeks after SCI (A and A′), dense glial processes from reactive astrocytes form a glial scar, preventing any *β*-tubulin–positive neurites from elongating through. (B–B′′) In contrast, after Fgf2 treatment long glial GFAP+ bridges have formed, which support the growth of long *β*-tubulin neurites alongside. Four months after SCI (C), similar scarring formed in PBS treatment and (D) some glia bridges formed in Fgf2 treatment. Scale bars in (A, B, C, and D) are 200 *μ*m, and in (A′, B′, and B′′) are 10 *μ*m. Fgf, fibroblast growth factor; SCI, spinal cord injury; PBS, phosphate buffered saline.

**Figure 6:**
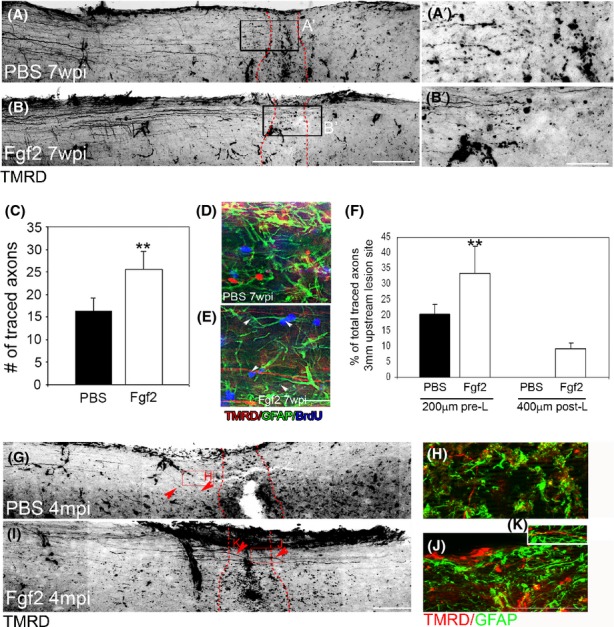
Fgf2 mediates axonal regeneration through the lesion site. Seven weeks postinjury (A and A′), anterograde tracing demonstrates that axons in PBS mice reach but do not enter the lesion, whereas regenerating axons in Fgf-treated mice enter and start to cross the lesion (B and B′); lesion site borders are marked with a red dotted line. (C) Quantitation of traced axons 100 *μ*m upstream to the lesion shows significantly more axons 2 weeks after SCI in Fgf2-treated mice (**P < 0.001, PBS *n* = 8; Fgf2 *n* = 10). Triple labeling of BrdU (blue), GFAP (green), and TMRD (red) in (F) PBS or (G) Fgf2 treatments. (F) Quantitation of traced axons 200 *μ*m upstream to the lesion and 400 *μ*m downstream to the lesion site 4 months after SCI shows that in Fgf2 treatment axons are crossing through the lesion site in oppose to PBS (**P < 0.001, PBS *n* = 4; Fgf2 *n* = 4). (G and I) Anterograde tracing demonstrates that axons in PBS mice are not crossing the lesion site; however, they are crossing in Fgf2-treated mice. (H, J, and K) Double-labeled TMRD (red) with GFAP (green) show that in PBS (H), astrocyte processes are going to random directions and do not support traced axons, whereas in Fgf2 (J and K) axonal processes are located along GFAP processes. Scale bars in (A, B, G, and I) are 200 *μ*m, in (A′ and B′) are 50 *μ*m, and in (D, E, H, J, and K) are 25 *μ*m. Fgf, fibroblast growth factor; PBS, phosphate buffered saline; SCI, spinal cord injury.

In control animals at 7 weeks post-SCI (with two first week of Fgf2/PBS treatment), reactive GFAP-positive astrocytes formed a glial scar, characterized by dense networks of processes around and at the lesion 7 weeks post-SCI. Although *β*-tubulin–labeled neurites are present within the lesion, they do not extend through the dense network of glial processes (Fig. 5A and A′). Fgf2 treatment for the first 2 weeks after injury induced a bipolar morphology within GFAP-positive cells, enabling neurites from neighboring neurons to grow along elongated glial processes, and consequently long *β*-tubulin–labeled can be seen extending through the lesion site (Fig. 5B and B′). Although gliosis and overall GFAP expression is lower in the Fgf2-treated mice, more of the GFAP-positive processes contribute to these parallel bridges (Fig. 5A and B). We saw the same result 4 months after SCI (Fig. 5C and D). These results are similar to what previously has been seen in zebrafish (Goldshmit et al. 2012) and suggests that Fgf2 drives changes in glial morphology to bridge the gap of the lesioned area and support neurite regeneration through the lesion. To test this we next investigated the effect of Fgf2 treatment on regeneration of descending neuronal tracts. To undertake this analysis, we injected the anterograde tracer TMRD at 6 weeks or 4 months postinjury, at the cervical level, upstream of the lesion of the 2-week treatment group. Treatment with Fgf2 resulted in a significant increase in the number of axons upstream to the lesion site 7 weeks after injury (Fig. 6A–C; 100 *μ*m upstream to the lesion). Additionally, a small proportion of axons entered and started to cross the injury site in Fgf2-treated mice only (Fig. 6B and B′). Triple labeling showed that astrocyte processes (GFAP positive) of proliferative cells (BrdU positive) were often aligned parallel to and along regenerating axons (tracer labeled) in Fgf2-treated animals in contrast to processes in PBS-control mice, which were oriented more randomly (Fig. 6D and E arrowheads). Thus, Fgf2 mediates astrocyte bipolar bridge morphology, which subsequently supports the growth of regenerating axons through the lesion site. Furthermore, tracer injection upstream of the lesion site 4 months postinjury shows that 10% of labeled axons cross the lesion site after Fgf2 treatment compared to control where no labeled axons cross (Fig. 6F–J, arrowheads). In addition, the GFAP-expressing astrocytes do not block axons, but are instead arranged parallel to the regenerating axons at the lesion site, whereas in PBS-control their direction is more random (Fig. 6H, J, and K).

### Fgf2 increases neurogenesis at the lesion site

**Figure 7:**
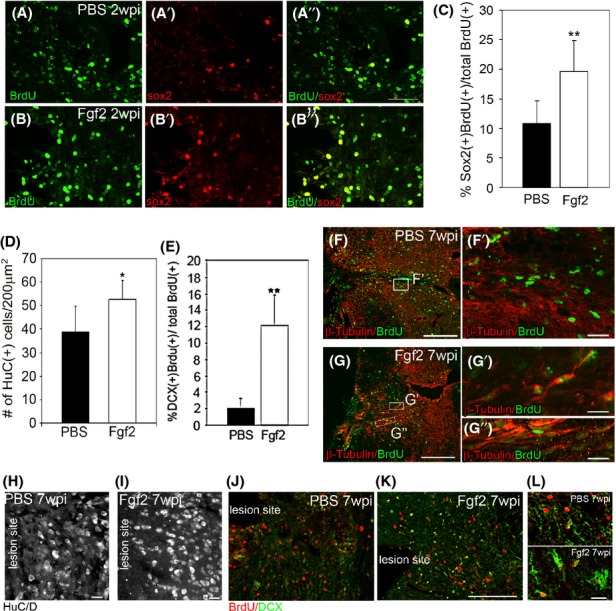
Fgf2 mediates neurogenesis. Two weeks after SCI, (A–A′′) sox2/BrdU double-positive cells at the lesion site gray matter in PBS control are lower (*n* = 5) than (B–B′′) after Fgf2 treatment, (*n* = 5). (C) Significantly higher percentage of sox2-positive cells proliferated at the lesion site after Fgf2 treatment (**P < 0.001). Seven weeks after SCI (D), quantitation of total neuronal cells (HuC/D+) from both sides shows significantly more neurons after Fgf2 treatment (*P < 0.05, *n* = 3 per group). (E) Quantitation of DCX+/BrdU+ cells shows significant increases in neurogenesis after Fgf2 treatment (**P < 0.001, *n* = 3 per group). Double labeling of BrdU (green) and *β*-tubulin reveals long outgrown neurites of newly born neurons after Fgf2 treatment (G), which are not present after PBS treatment (F). (H) An example of HuC/D staining in PBS-control lesion site, (I) an example of HuC/D staining in Fgf2-treated animal lesion site, (J) an example of DCX/BrdU staining in PBS-control lesion site, (K) an example of DCX/BrdU staining in Fgf2-treated animal lesion site, (L) upper-panel high-power magnification of DCX/BrdU staining in PBS-control lesion site, and lower-panel high-power magnification of DCX/BrdU staining in Fgf2 lesion site. Graphs show mean axons or cell number/field ±SEM. Scale bars in (A–A′′ and B–B′′) are 100 *μ*m, in (F′, G′, G′′, H, I, and L) are 10 *μ*m, and in (F, G, J, and K) are 200 *μ*m. Fgf, fibroblast growth factor; SCI, spinal cord injury; PBS, phosphate buffered saline; DCX, doublecortin.

Two weeks after injury, Sox2, a transcription factor that regulates neuronal stem cells during central nervous system development (Ellis et al. 2004; Fong et al. 2008), was significantly increased after Fgf2 treatment within the gray matter at the lesion (Fig. 7A–A′′, B–B′′, and C). In a later time point, at 7 weeks postinjury, Fgf2 treatment also significantly increased neurogenesis at the lesion (Fig. 7D–G). Quantitation from both sides of the lesion showed a significant increase in the total number of the postmitotic neuronal marker HuC/D in Fgf2-treated (52.4 ± 8.1) compared to compared to PBS-control mice (38.6 ± 11.0, Fig. 7D, H, and I). A significantly higher percentage of newborn BrdU-positive cells expressed the early neuron marker DCX in Fgf2-treated (12.1 ± 3.69) compared to PBS-control mice (2.0 ± 1.28, Fig. 7E and J–L). Colabeling of BrdU with the cytoskeletal neuronal marker *β*-tubulin only revealed double-labeled neurons with elongated *β*-tubulin–positive processes in Fgf2-treated mice, whereas BrdU + /*β*-tubulin + cells could not be confidently identified in the PBS-control mice (Fig. 7F–F′ and G–G′′). Thus, Fgf2 contributes to both neurogenesis as well as neuronal cell survival.

## Discussion

**Figure 8:**
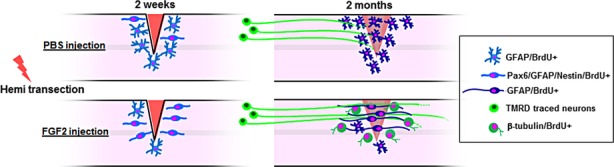
Model for fibroblast growth factor (Fgf)2-mediating glia bridges after spinal cord injury in mouse. Fgf2 increases neurogenesis and radial/progenitor cell marker expression and mediates polarized morphology of glial cells which form glia bridges that support axonal regeneration through the lesion.

Here, we show that Fgf2 treatment after SCI in mice results in a reduced inflammatory response and decreased astrocyte reactivity and glial scar formation. Glial scarring was reduced not only by decreasing the number of glia and glial processes but also by reducing levels of cytokine and CSPGs at the lesion and monocyte/macrophage infiltration. Moreover, increased Fgf2 signaling at the lesion site promoted the formation and propagation of radial/progenitor bipolar glia cells which at later stages mediated the formation of GFAP-expressing glial bridges that support regenerating neuronal processes to traverse the lesion (Fig. 8). Together, these effects create a permissive environment for regeneration at the lesion site and stimulating glia to generate new progenitors. The similarity to the Fgf-dependent mechanisms evident in zebrafish post-SCI, a proregenerative model, is striking and suggests that distinct regulation of Fgf signaling mediates the differential regenerative capacity of the two systems. In both cases the major cell population that responds to the injury by proliferation and migration to the lesion site are the GFAP-positive glial cells. In addition to reactive astrocytes, diverse stem and progenitor cell populations are activated after SCI in rodents (Meletis et al. 2008; Petit et al. 2011). However, these cell populations are non-neurogenic under normal physiological or pathological conditions in the mammalian spinal cord. As a result, a glial scar composed of dense processes is formed, which prevents neurite regeneration through the lesion in murine SCI. Our work shows that addition of exogenous Fgf2 after SCI in the mouse spinal cord has several important proregenerative effects. First, reactive proliferating astrocytes dedifferentiate to increase radial glia numbers at the lesion (Yang et al. 2011), second, the existing population of radial glia within the spinal cord start proliferating. In agreement with this result we show that Pax6-positive, Sox2-positive, and nestin-positive cells in PBS-injected animals remain low within the gray matter after SCI. In contrast, Fgf2-treated mice show a significant increase in cells that colabel with all three markers 2 weeks after injury. The change in marker expression is accompanied by changes in glial cell morphology and behavior. Fgf2 treatment shifts the glial population from cells with astroglial morphology toward cells with radial and bipolar morphology. Similarly, Fgf signaling changes glia morphology in the zebrafish spinal cord (Goldshmit et al. 2012) or in mammalian astrocytes in vitro (Imura et al. 2006; Goldshmit et al. 2012; Lichtenstein et al. 2012). The radial and bipolar glia cells promote the formation of bridges that support axonal regeneration through the lesion. Furthermore, Fgf2 injection increases neurogenesis and neuronal survival consistent with previous reports (Meijs et al. 2004). Importantly, we show functional improvement in behavioral assays 5 weeks post-SCI in Fgf2-treated mice, consistent with other studies in rodents (Lee et al. 1999; Rabchevsky et al. 1999).

We further suggest that the GFAP-expressing cells that coexpress progenitor markers, such as nestin and Pax6, may be responsible for the ability to form bridges at the lesion site and supply a proregenerative conditions for future axonal regeneration that was observed at a later time point of 4 months. Although a significantly higher number of axons had grown and reach the lesion at 7 weeks after injury in Fgf2-treated mice, only a few axons had actually traversed the lesion site. Therefore, we believe that the observed functional improvements are more likely caused by the reduced scarring, enhanced neurogenesis, and survival of neurons detected during the first weeks after injury than axonal regeneration and reconnection.

Given that modest improvement can be achieved in chronic patients treated with Fgf1 (Wu et al. 2008), after removing the scar and suppling locally the Fgf in a biological glue, our results suggest that application of Fgf at an acute injury phase may lead to a significantly enhanced functional recovery in humans through the modulation of glial scar formation.
